# Resignation

**Published:** 1859-02

**Authors:** 


### RESIGNATION.

Prof. H. A. Johnson has resigned the chair of Physiology and Pathology in Rush Medical College. Prof. J. was an able and interesting lecturer; always highly esteemed by the classes attending the college; and his resignation has been received with much regret by his colleagues in the faculty.

